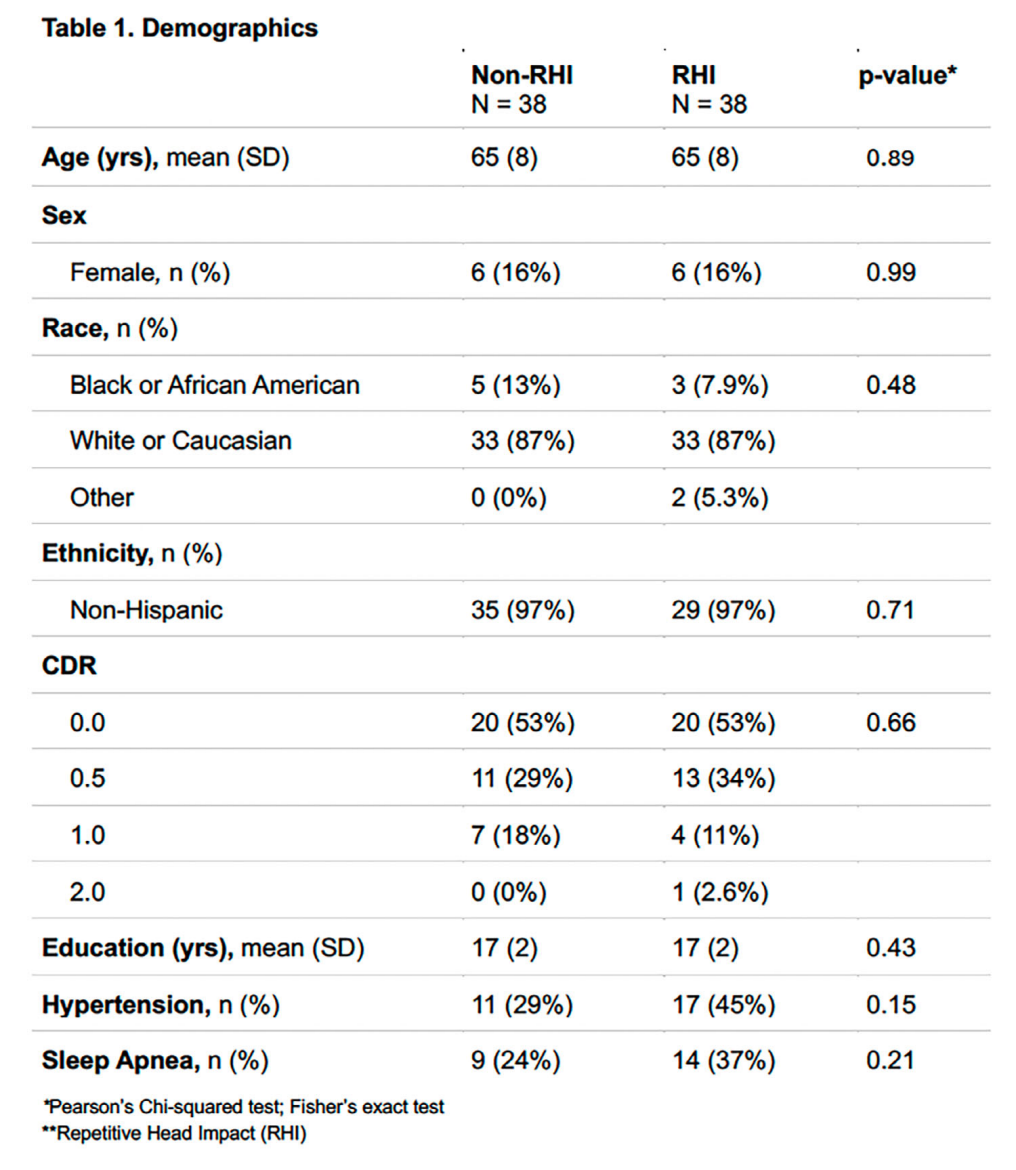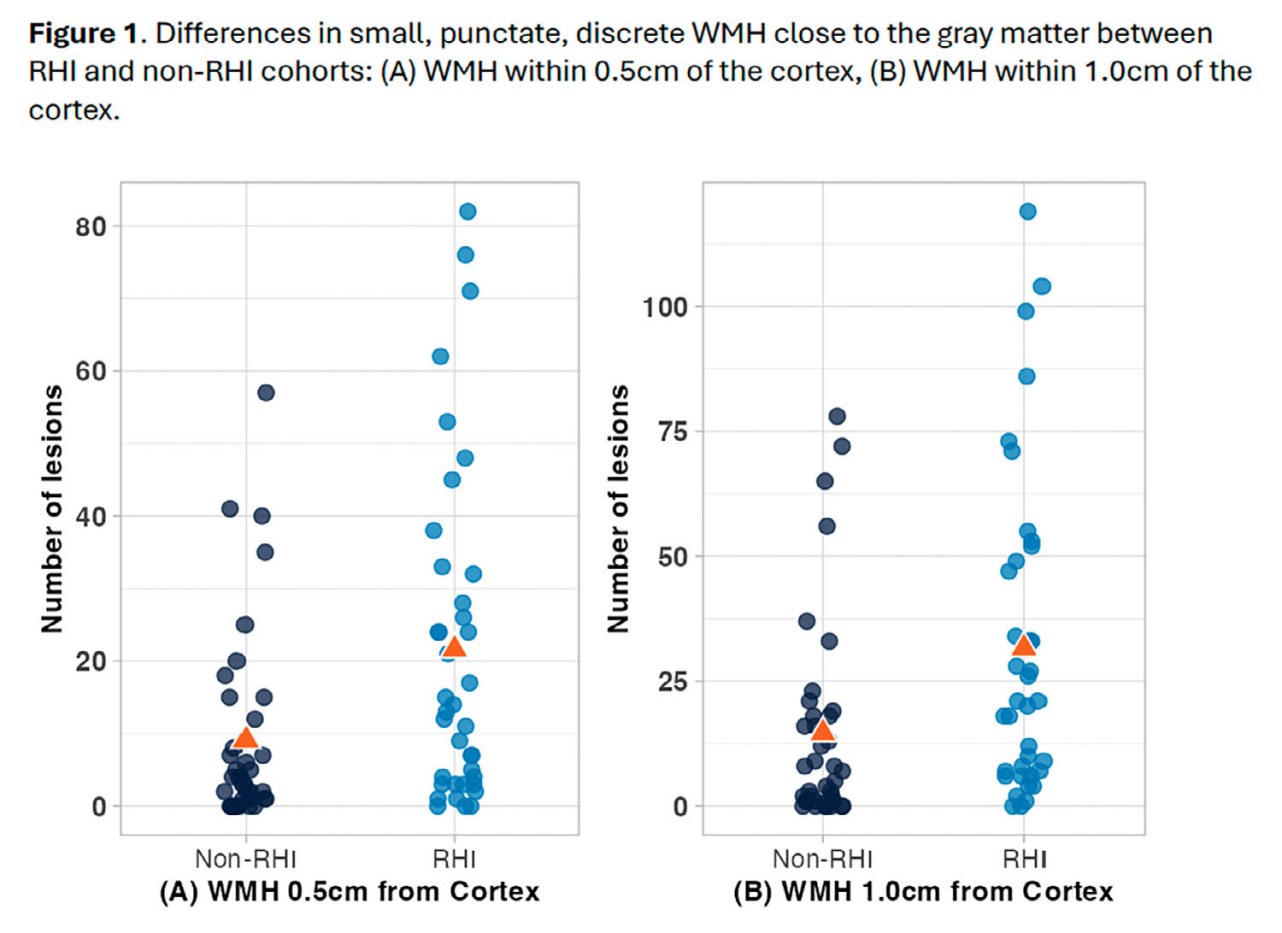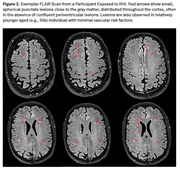# Unique Pattern of White Matter Hyperintensities in Middle Age and Older Adults with History of Repetitive Head Impact Exposure

**DOI:** 10.1002/alz.091572

**Published:** 2025-01-09

**Authors:** Jenna R. Groh, Annalise E. Miner, Chad Farris, Yorghos Tripodis, Christopher J. Nowinski, Anna Cui, Erika C. Pettway, Fatima Tuz‐Zahra, Mohamad J. Alshikho, Adam M. Brickman, Steven Lenio, Monica T. Ly, Caroline Altaras, Andrew E. Budson, Katherine W. Turk, Wendy Qiu, Lee E Goldstein, Brett Martin, Joseph N. Palmisano, Eric G. Steinberg, Robert A. Stern, Thor D. Stein, Ann C. McKee, Jesse Mez, Michael L. Alosco

**Affiliations:** ^1^ Boston University Chobanian & Avedisian School of Medicine, Boston, MA USA; ^2^ Boston University Alzheimer’s Disease Research Center, Boston, MA USA; ^3^ Boston Medical Center, Boston, MA USA; ^4^ Boston University School of Public Health, Boston, MA USA; ^5^ Concussion Legacy Foundation, Boston, MA USA; ^6^ Taub Institute for Research on Alzheimer's Disease and the Aging Brain, New York, NY USA; ^7^ Columbia University Irving Medical Center, New York, NY USA; ^8^ Department of Neurology, Vagelos College of Physicians and Surgeons, Columbia University, and the New York Presbyterian Hospital, New York, NY USA; ^9^ VA Boston Healthcare System, Jamaica Plain, MA USA; ^10^ VA Bedford Healthcare System, Bedford, MA USA

## Abstract

**Background:**

Repetitive head impacts (RHI) from contact sports can lead to long‐term white matter injury visualized on FLAIR scans as white matter hyperintensities (WMH). The goal of this study was to preliminarily characterize the unique pattern and features of WMH in middle aged‐ to older adults with remote history of exposure to RHI from contact sports.

**Method:**

76 participants (38 with substantial RHI, 38 with minimal or no RHI) from the Boston University Alzheimer’s Disease Research Center had a FLAIR MRI during their annual study visit. The presence of moderate‐severe WMH was adjudicated by a panel of clinicians (including of a neurologist and neuroradiologist) during a multidisciplinary diagnostic consensus. Previously, we observed small, spherical, discrete lesions proximal to the gray matter in individuals exposed to RHI. Therefore, the number of discrete, spherical WMH within 1.0cm of the cortex was counted. Regression and analysis of variance models examined group effects as well as years of American football play (proxy for duration of RHl), controlling for age at MRI and vascular risk factors.

**Results:**

Sample characteristics are in Table 1. Sources of RHI were American football, hockey, soccer, wrestling, field hockey, lacrosse, mixed martial arts, and rugby. The RHI group was judged to have greater burden of WMH compared to non‐RHI (n=15 vs n=5, p=0.042). Years of football play was associated with greater odds for having moderate‐severe WMH (OR =1.10, p=0.023). RHI participants had a greater number of unique, distinguishable WMH within 0.5cm (p=0.006) and 1.0cm (p=0.008) of the cortex (Figure 1). Qualitatively, we visualized small, punctate lesions close to the gray matter, clustered around the depths of the sulci in the RHI cohort, distinct from the patterns typically seen with chronic ischemic disease and neuro‐inflammatory conditions (Figure 2).

**Conclusion:**

We propose RHI induces a unique pattern of WMH characterized by small, spherical, punctate lesions proximal to the deep gray matter distributed throughout the cerebrum. The lesion location corresponds to areas susceptible to RHI and p‐tau in chronic traumatic encephalopathy. Future research targets include quantitative analysis to better characterize features and patterns of WMH, clarify specificity to RHI, and study biological correlates.